# Supplementary material for: Nanosecond pulsed electric fields induce cell-size-dependent selective permeabilization of urothelial cancer cells
Source: Commun Biol. 2025 Dec 30;9:153. doi: 10.1038/s42003-025-09432-7 (PMC12868620; doi:10.1038/s42003-025-09432-7)
Supplement: Supplementary file 2 — Supplementary Information [file 42003_2025_9432_MOESM2_ESM.pdf]

Exposure to Nanosecond Pulsed Electric Fields Reveals Increased Cell Membrane Vulnerability and Impaired cell membrane Repair in Urothelial Cancer Cells

Aleksander Kielbik[1], Emily Hellwich[2], Veronika Bahlinger[3], Pamela Sowa[4], Daniel Lambton[2], Markus Kühs[1], Maria Luisa Barcena[1], Olesya Vakhrusheva[1], Hendrik Proebsting[5], Simon Walz[1], Tilman Schäffer[2], Falko Fend[3], Vitalij Novickij[6,7], Bastian Amend[1], Igor Tsaur[1]

1. Department of Urology, University Hospital Tuebingen, Tuebingen, Germany
2. Institute of Applied Physics, University of Tuebingen, Tuebingen, Germany
3. Institute of Pathology and Neuropathology and Comprehensive Cancer Center, University Hospital Tuebingen, Tuebingen, Germany
4. Department of Cardiology and Angiology, University Hospital Tuebingen, Tuebingen, Germany
5. University Hospital Tuebingen, Faculty of Medicine, Eberhard Karls University Tuebingen, Germany
6. Institute of High Magnetic Fields, Vilnius Gediminas Technical University, Vilnius, Lithuania,
7. Department of Immunology and Bioelectrochemistry, State Research Institute Centre for Innovative Medicine, Vilnius, Lithuania

*corresponding author: aleksander.kielbik@med.uni-tuebingen.de

Table of content

Content	Description	Page
Supplementary Figure **S1**	**Finite element simulation of electric field distribution and design of a high-frequency electroporator for experimental applications**	3
Supplementary Figure **S2**	**Voltage-dependent changes in final YO-PRO-1 fluorescence and fluorescence changes after sham exposure**	4
Supplementary Figure **S3**	**YO-PRO-1 fluorescence changes in 3-second intervals following exposure to nsPEFs.**	5
Supplementary Figure **S4**	**Cell size evaluation in tissue microarrays containing normal urothelial tissue, primary urothelial carcinoma, and lymph node metastases and in sections of PDO and spheroids**	6-8
Supplementary Table **S1**	**Clinical data of patients whose tumor tissue was used to create PDOs.**	9
Supplementary Figure **S5**	**Histological evaluation of the patient derived oganoids (PDO)**	10-11
Supplementary Figure **S6**	**Image analysis pipeline for spheroid electroporation**	12
Supplementary Figure **S7**	**YO-PRO-1 fluorescence Intensity and area under the curve 10 minutes after nsPEFa exposure of spheroids. Time course of fluorescence following sham exposure.**	13-14
Supplementary Figure **S8**	**Changes in the 2D projected area of spheroids and PDOs following exposure to nsPEFs.**	15
Supplementary Figure **S9**	**Overview over technical workflow.**	16
Supplementary Figure **S10**	**Image analysis pipeline for spheroid electroporation**	17

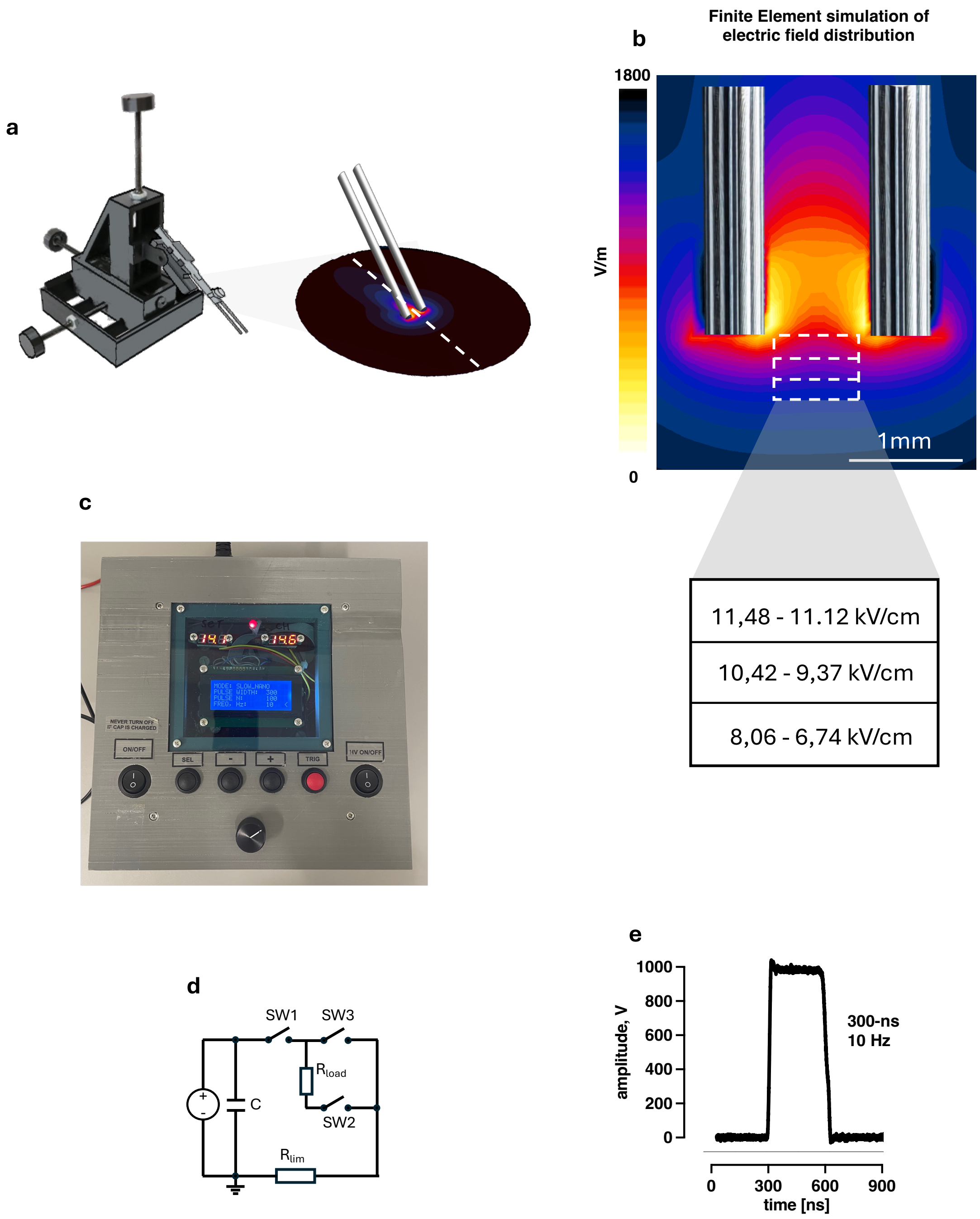

Supplementary Figure S1: Finite Element Simulation of Electric Field Distribution and Design of a High-Frequency Electroporator for Experimental Applications. a. A custom-made electrode, consisting of two tungsten rods, was positioned at a fixed 45-degree angle to the cell monolayer using a 3D-printed micromanipulator. **b.** Electric field distribution was simulated using the finite element solver S4Llite, with 1 volt applied across the electrodes. The dashed line in the simulation image marks the region of interest used for fluorescence image analysis. Electric field values were calculated by multiplying the simulated field by the actual voltage applied during electroporation. **c.** A custom-built pulse generator was designed to deliver a series of rectangular, nanosecond-duration electric pulses. **d.** Simplified scheme of the high-frequency electroporator used in this study. The load—comprising the physiological solution and cells positioned between the electrodes—is represented as the resistor R_{LOAD}. When both switches SW1 and SW2 are closed, a high-voltage pulse is delivered to the load. SW3 is a crowbar, to ensure constant load-independent fall time of the pulse. **e.** An example of a rectangular pulse generated by the pulser.

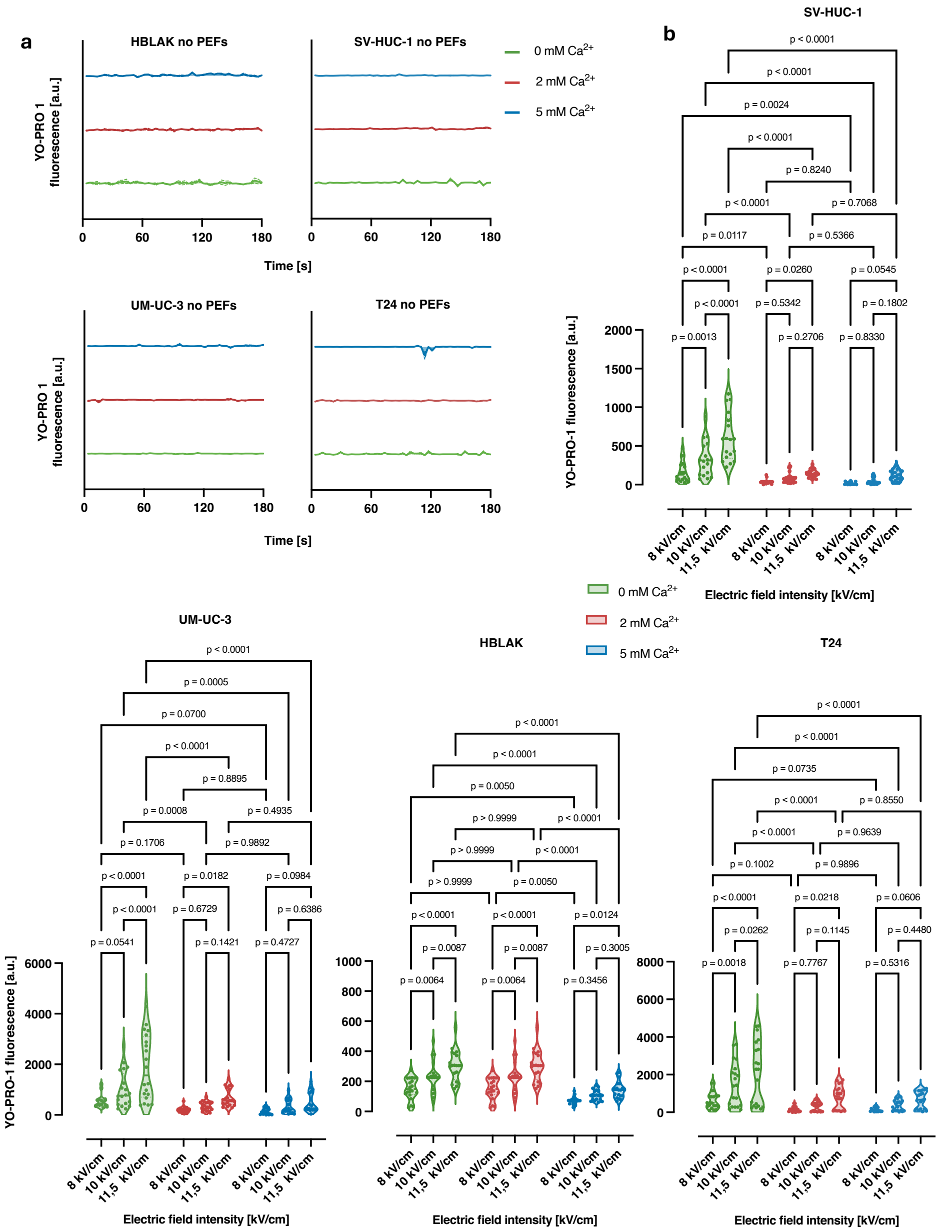

Supplementary Figure S2: Voltage-dependent changes in final YO-PRO-1 fluorescence and fluorescence changes after sham exposure a. Time course of YO-PRO-1 fluorescence in monolayers incubated in solutions containing 0 mM, 2 mM, or 5 mM Ca^{2+} without exposure to nsPEFs, shown as mean ± s.e. Traces were vertically offset along the y-axis for improved visual clarity. All measurements originated from the same baseline fluorescence intensity, and the vertical displacement does not reflect differences in absolute fluorescence values. No detectable YO-PRO-1 uptake was measured in sham-exposed monolayers during the observation period, regardless of cell line or Ca^{2+} concentration. **b.** Violin plot showing the distribution of mean YO-PRO-1 fluorescence intensity in monolayers of HBLAK, SV-HUC-1, UM-UC-3, and T24 cell lines, measured 180 seconds after exposure in solutions containing 0 mM, 2 mM, and 5 mM Ca^{2+} at electric field strengths of 11.5 kV/cm, 10 kV/cm, and 8 kV/cm (n = 18–20). Two-way ANOVA with Geisser–Greenhouse correction and Tukey's post hoc test for multiple comparisons was performed. For HBLAK cells, the electric field factor yielded $F(2, 171) = 37.88$, and the Ca^{2+} factor $F(2, 171) = 46.38$. In UM-UC-3 cells, the electric field resulted in $F(2, 165) = 25.03$ and Ca^{2+} in $F(2, 165) = 37.17$. For SV-HUC-1 cells, the electric field factor was $F(2, 161) = 37.84$, and the Ca^{2+} factor $F(2, 161) = 87.15$. In T24 cells, the electric field produced $F(2, 171) = 20.43$ and Ca^{2+} $F(2, 171) = 35.45$.

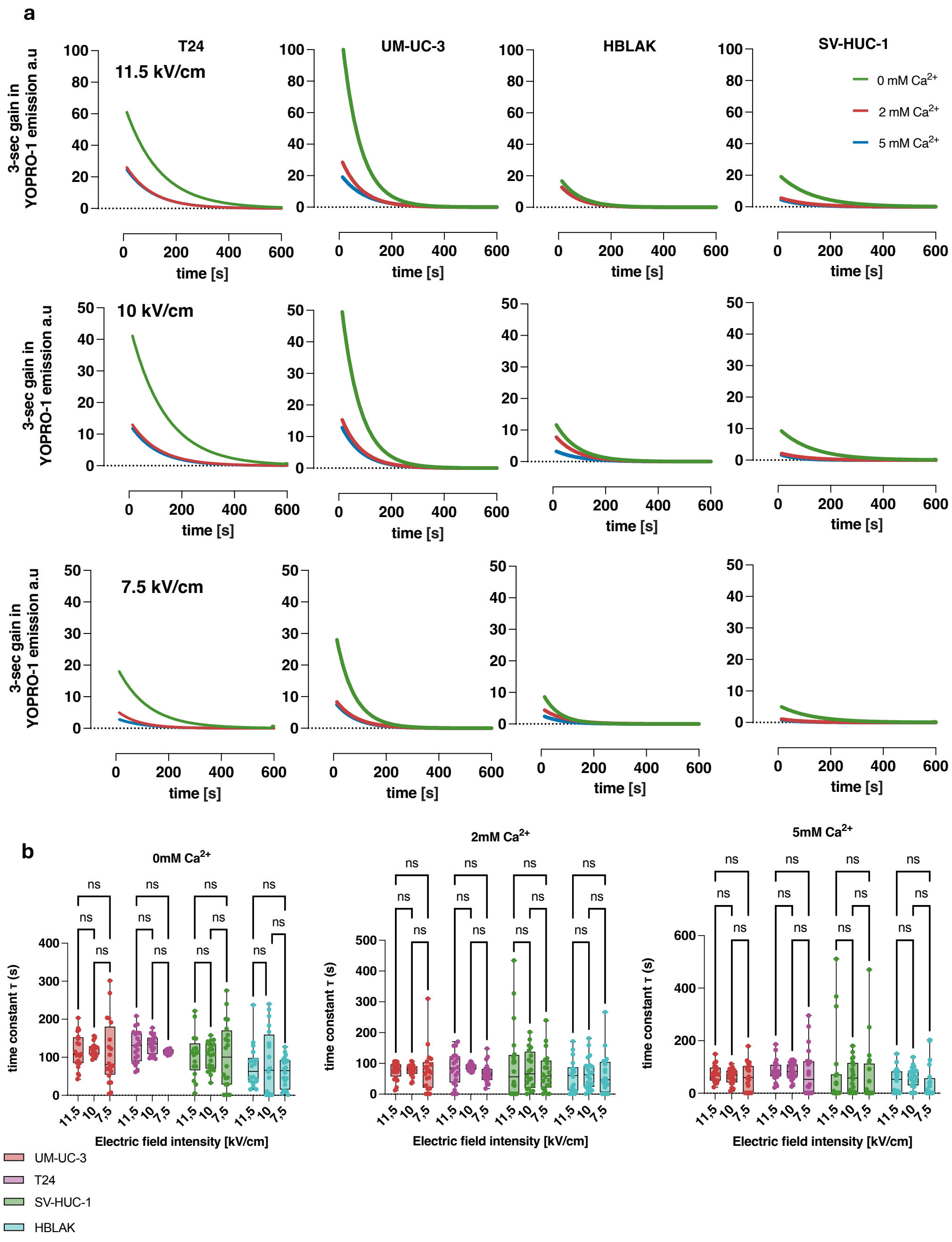

Supplementary Figure S3. a. YO-PRO-1 fluorescence changes in 3-second intervals following exposure to nsPEFs. Single-exponential curve of fluorescence decay following nsPEF exposure using data recorded in 3-second intervals after the pulse train. The presence of extracellular Ca^{2+} enhanced membrane resealing in urothelial cells. Distinct differences in membrane repair kinetics were observed between non-cancerous cell lines (SV-HUC-1 and HBLAK) and cancerous urothelial cell lines (T24 and UM-UC-3). The Ca^{2+}-dependent acceleration of membrane repair was particularly pronounced in the cancer cell lines. **b.** displays the average time constants derived from the exponential fits, shown as mean ± SEM (n = 18–20). Two-way ANOVA with Geisser–Greenhouse correction and Tukey's post hoc test for multiple comparisons was performed. In 0 mM Ca^{2+}, the electric field factor yielded $F(2, 209) = 1.011$ and the cell line factor $F(3, 209) = 10.08$. In 2 mM Ca^{2+}, the electric field factor was $F(2, 207) = 0.3248$ and the cell line factor $F(3, 207) = 0.7707$. In 5 mM Ca^{2+}, the electric field factor yielded $F(2, 219) = 0.4502$, and the cell line factor $F(3, 219) = 1.985$. No significant differences in τ were observed across electric field intensities in media containing 0 mM, 2 mM, or 5 mM Ca^{2+}.

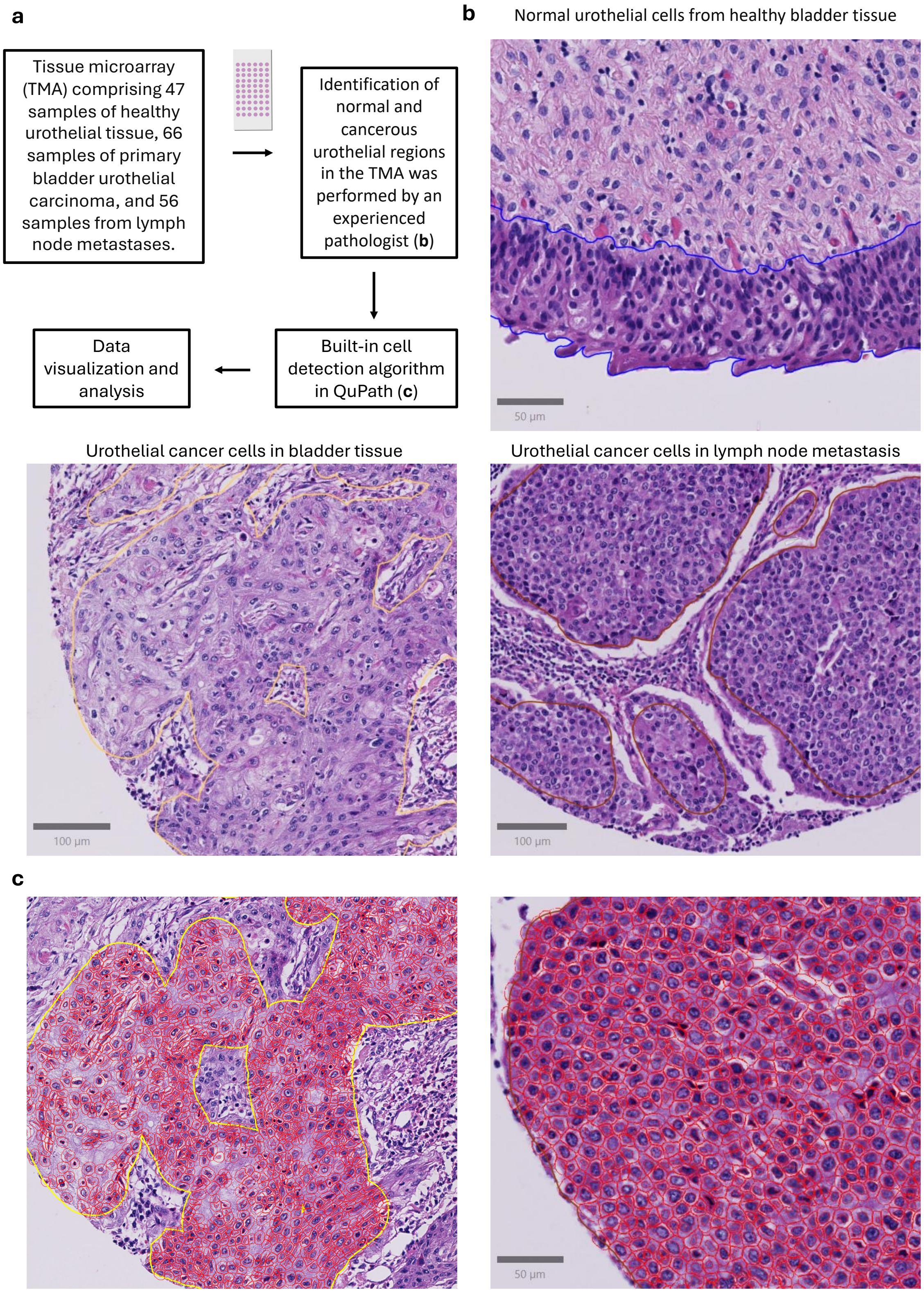
a
Tissue microarray (TMA) comprising 47 samples of healthy urothelial tissue, 66 samples of primary bladder urothelial carcinoma, and 56 samples from lymph node metastases.
Identification of normal and cancerous urothelial regions in the TMA was performed by an experienced pathologist (b)
Built-in cell detection algorithm in QuPath (c)
Data visualization and analysis
b
Normal urothelial cells from healthy bladder tissue
50 μm
Urothelial cancer cells in bladder tissue
100 μm
Urothelial cancer cells in lymph node metastasis
100 μm
c
50 μm

d Urothelial cancer cells in lymph node metastasis

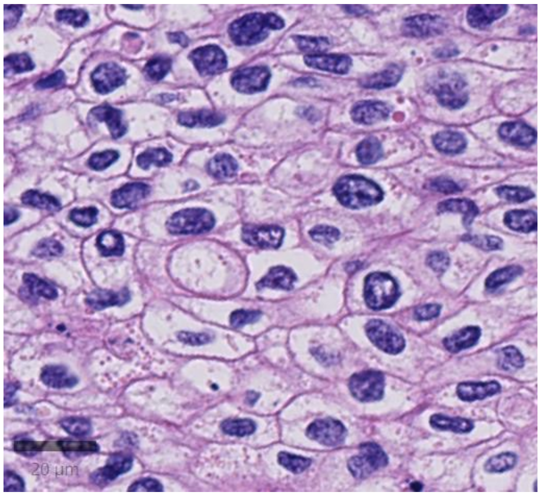

Urothelial cancer cells in bladder tissue

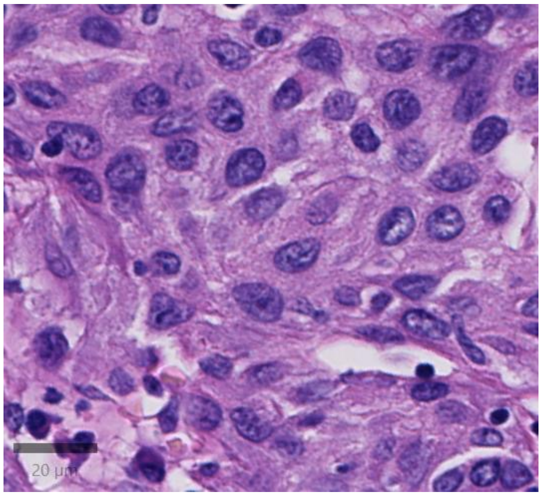

Normal urothelial cells from healthy bladder tissue

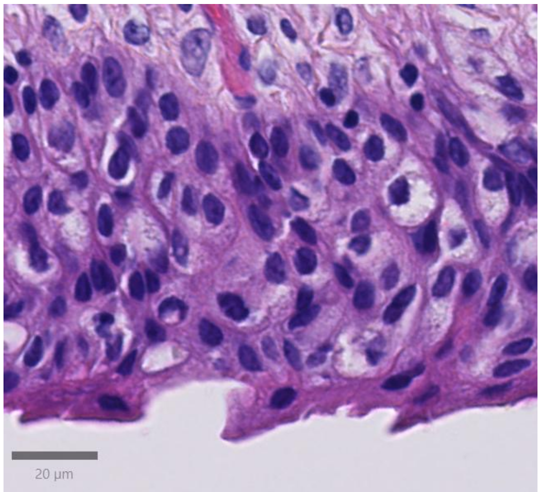

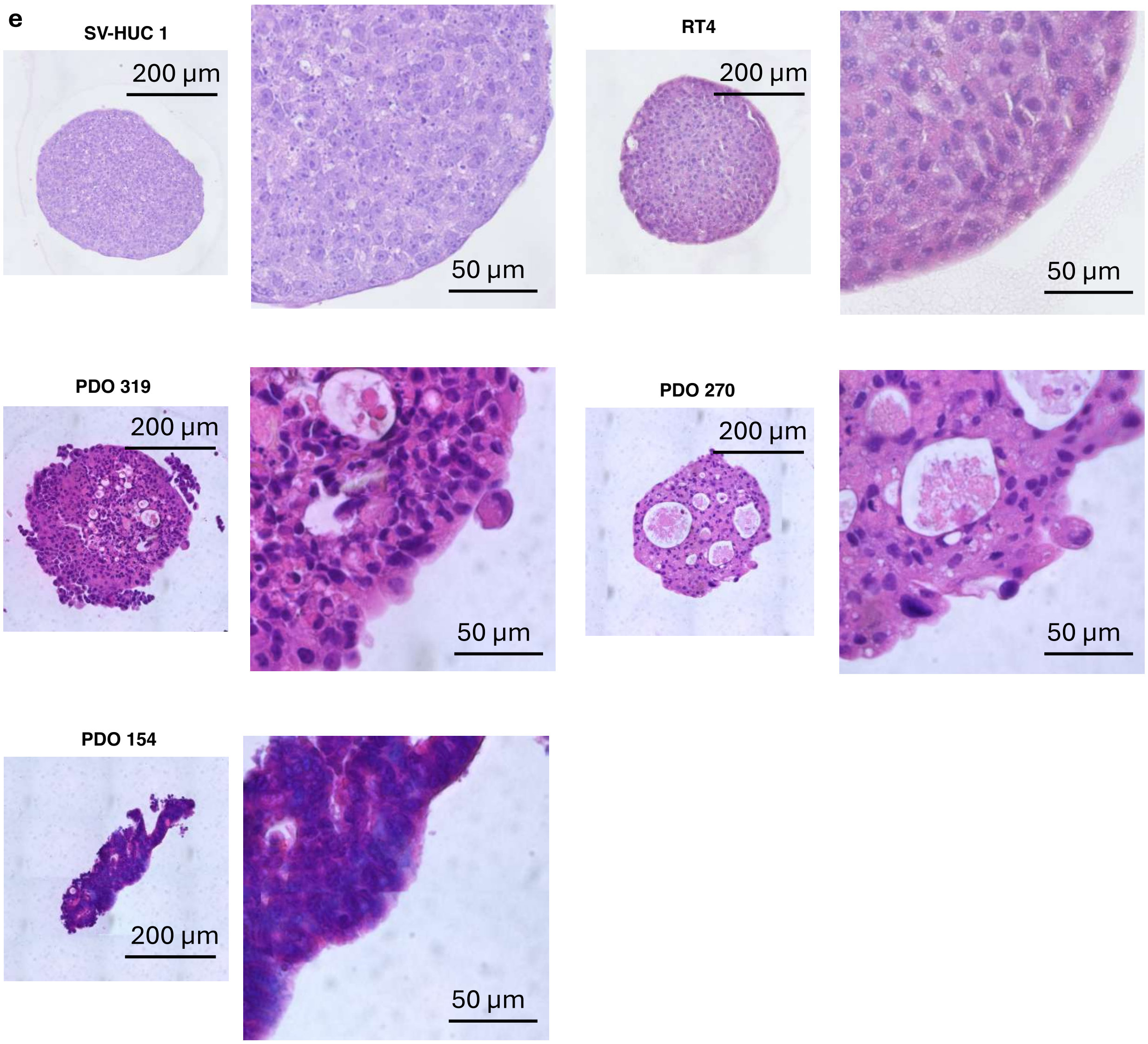

Supplementary Figure S4. Cell size evaluation in tissue microarrays containing normal urothelial tissue, primary urothelial carcinoma, and lymph node metastases. a. Schematic representation of the workflow for cell size analysis from the tissue microarray (TMA). b. Representative images of annotated tissue regions. c. The built-in cell detection algorithm in QuPath enables measurement of the projected 2D area of individual cells within the tissue section. d. High-magnification views of normal urothelial tissue and urothelial carcinoma tissue for visual comparison of cell morphology.e.

BCO ID	Stage	Tumor Type	Preoperative treatment	Surgical Treatmant
154	Locally advanced (≥pT3 or N+)	Small cell carcinoma	no	Radical surgery
270	Locally advanced (≥pT3 or N+)	Urothelial carcinoma	No	Transurethral resection
319	Locally advanced (≥pT3 or N+)	Urothelial carcinoma	Systemic therapy	Radical surgery

Supplementary Table S1. Clinical data of patients whose tumor tissue was used to create PDOs. Samples were obtained during radical cystectomy or transurethral resection of muscle-invasive bladder cancer. Following tissue processing, primary tumor cells were isolated and cultured to form spheroids suitable for electroporation studies. The resulting spheroids represent both invasive urothelial carcinoma and small cell carcinoma of the bladder.

a

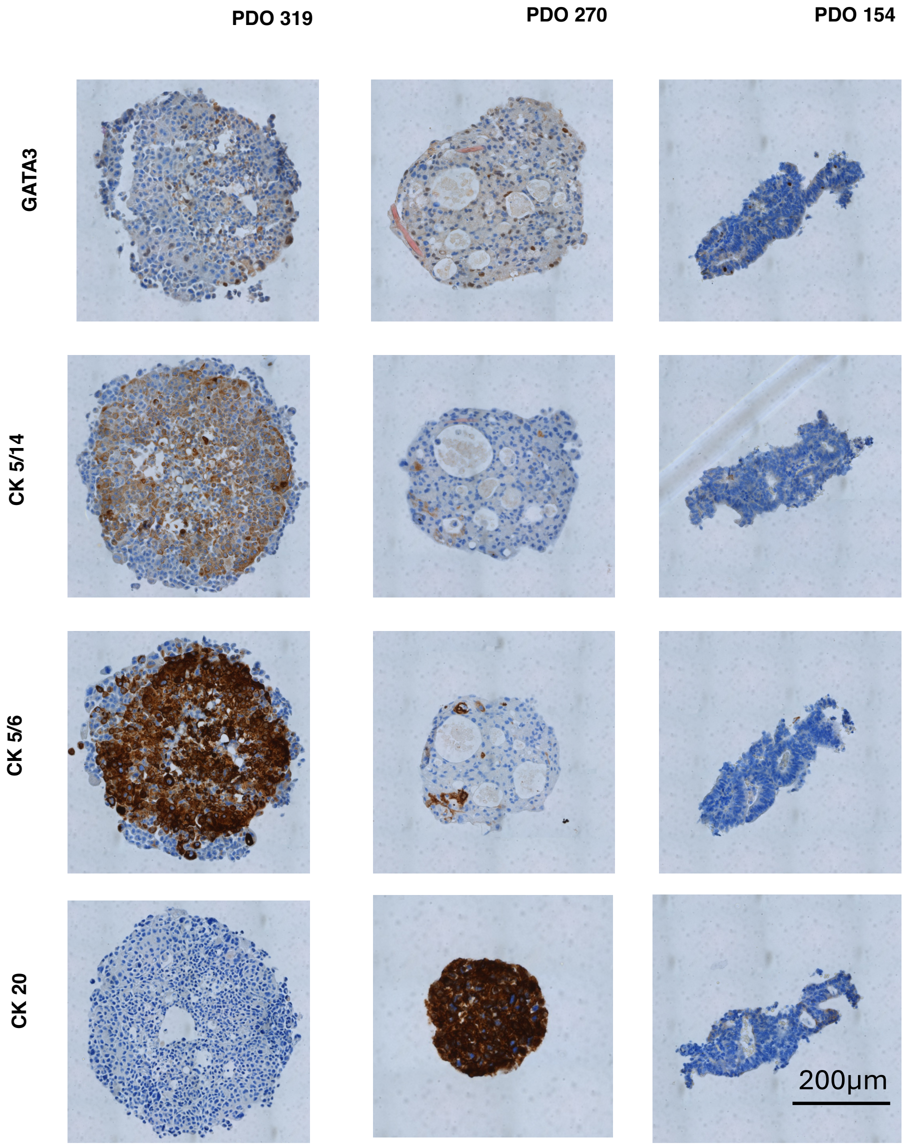

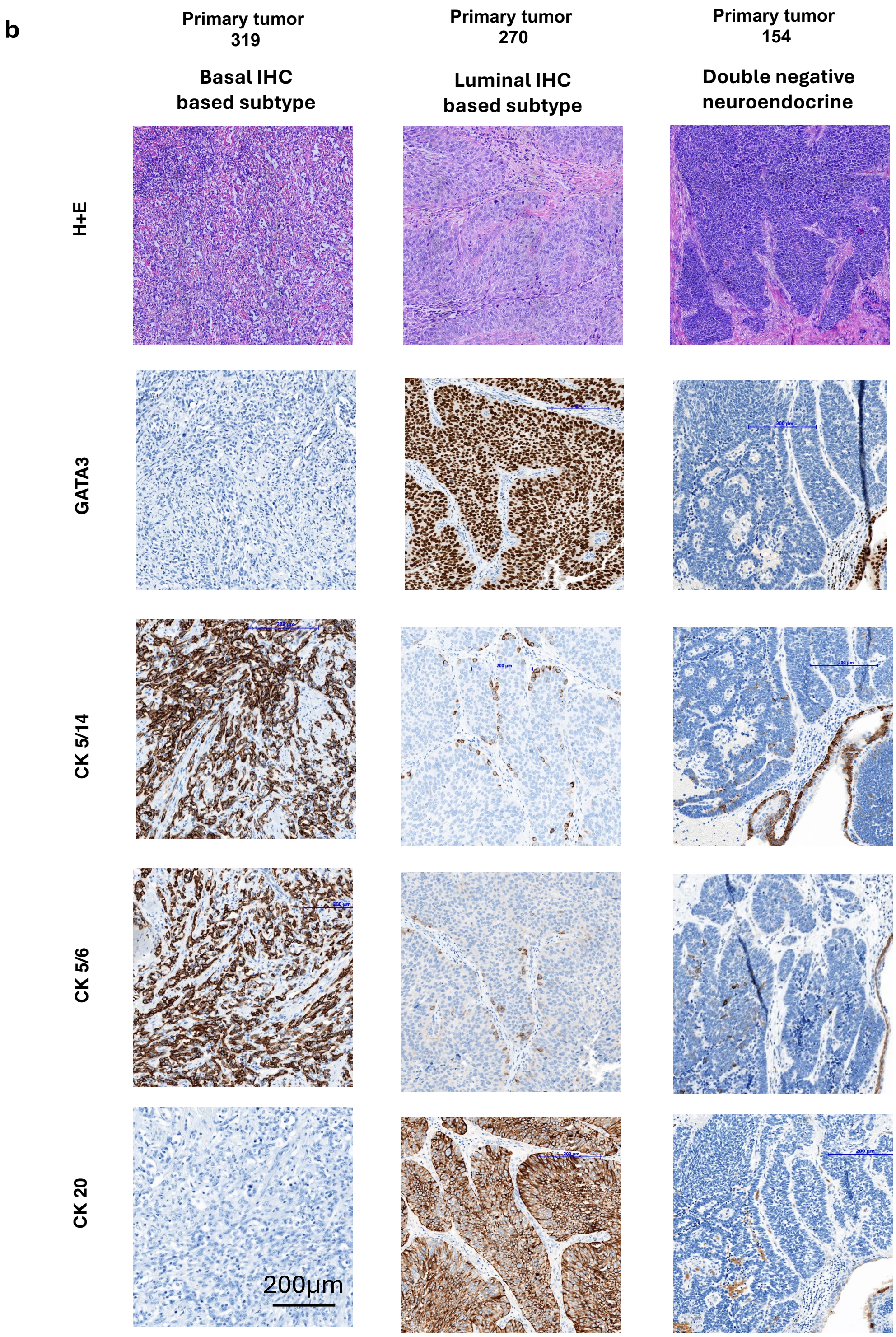

Supplementary Figure S5. Histological evaluation of patient-derived organoids (PDOs) and their corresponding primary tumor tissue. Cross-sections of PDOs 319, 270, and 154, along with matched tumor tissue, were stained with hematoxylin and eosin (H&E) and immunohistochemically labeled for GATA3, CK5/6, CK5/14, and CK20. The staining confirmed the urothelial origin of the primary cells within the organoids and demonstrated consistent expression of the tested markers.

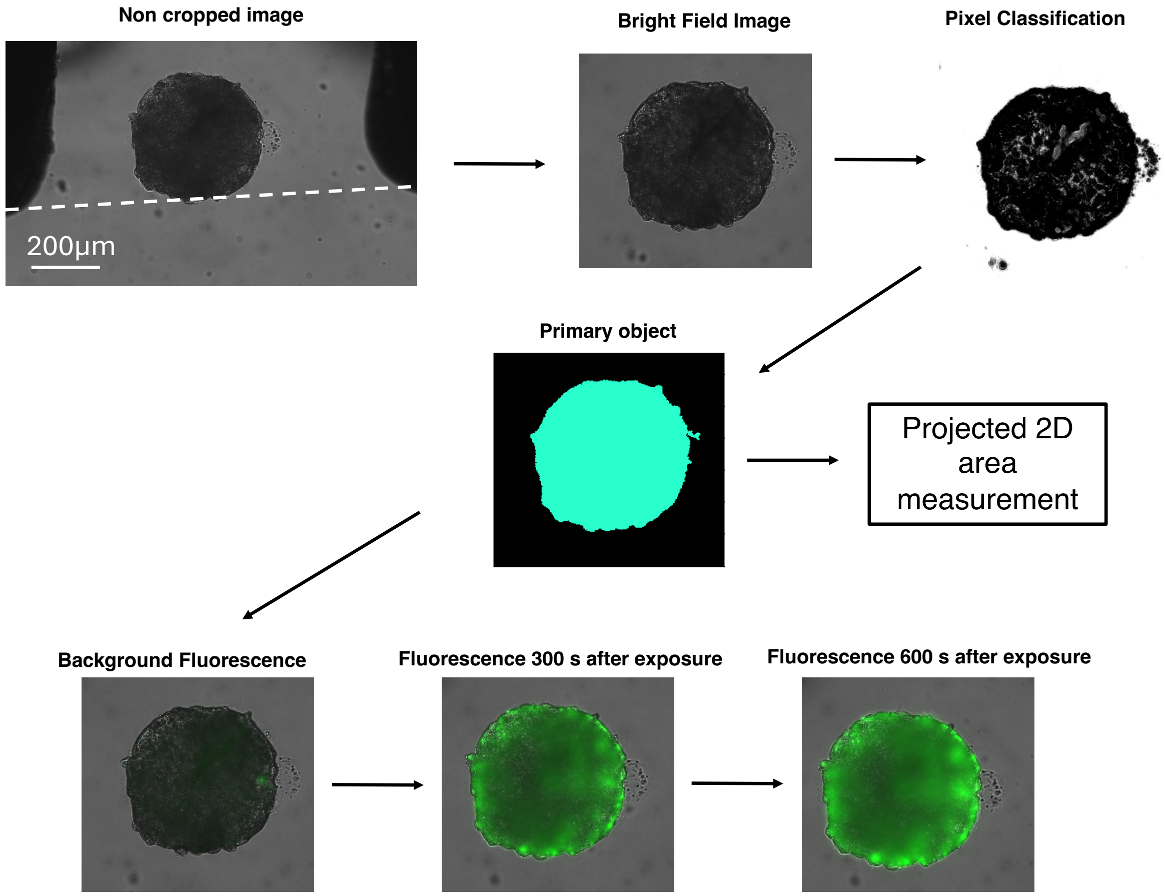

Supplementary Figure S6 Image analysis pipeline for spheroid electroporation. Machine learning–based pixel classification enabled the identification of the spheroid in each frame of the brightfield time-lapse sequence. Fluorescence changes were recorded over a 600-second period. The spheroid was defined as the primary object, and both its projected 2D area and internal fluorescence were tracked for 10 minutes following electroporation.

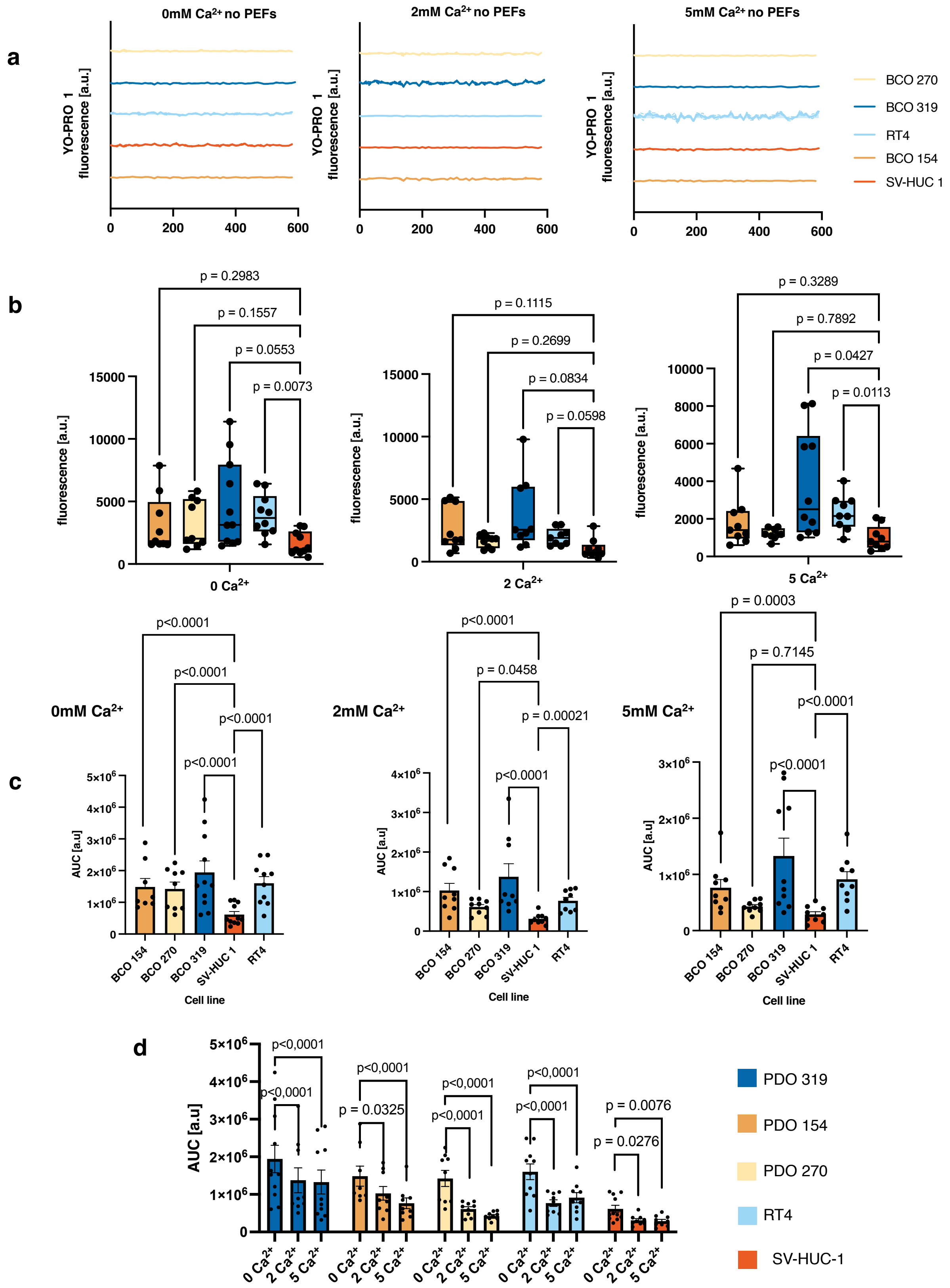
a
0mM Ca2+ no PEFs
2mM Ca2+ no PEFs
5mM Ca2+ no PEFs
YO-PRO 1 fluorescence [a.u.]
BCO 270
BCO 319
RT4
BCO 154
SV-HUC 1
b
p = 0.2983
p = 0.1557
p = 0.0553
p = 0.0073
fluorescence [a.u.]
0 Ca2+
p = 0.1115
p = 0.2699
p = 0.0834
p = 0.0598
2 Ca2+
p = 0.3289
p = 0.7892
p = 0.0427
p = 0.0113
5 Ca2+
c
0mM Ca2+
2mM Ca2+
5mM Ca2+
p<0.0001
p = 0.0458
p = 0.00021
p = 0.0003
p = 0.7145
AUC [a.u]
BCO 154
BCO 270
BCO 319
SV-HUC 1
RT4
Cell line
d
AUC [a.u]
p<0,0001
p = 0.0325
p = 0.0076
p = 0.0276
0 Ca2+
2 Ca2+
5 Ca2+
PDO 319
PDO 154
PDO 270
RT4
SV-HUC-1

e

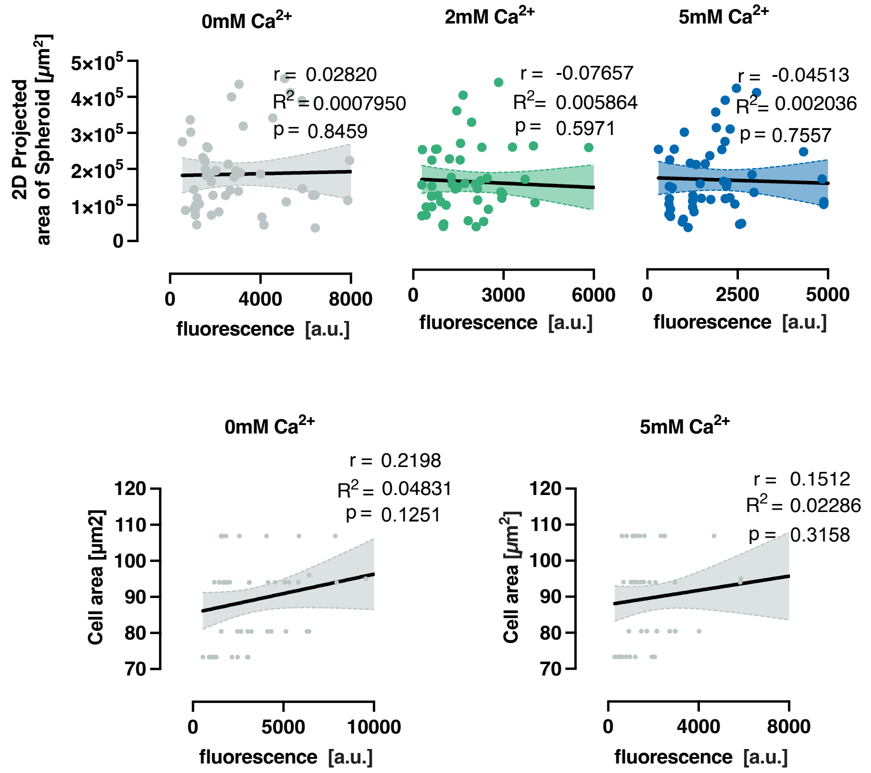

Supplementary Figure S7. YO-PRO-1 Fluorescence intensity and area under the curve (AUC) 10 Minutes after nsPEF exposure of spheroids, and time course of fluorescence following sham exposure. a. Time course of YO-PRO-1 fluorescence spheroids and PDOs incubated in solutions containing 0 mM, 2 mM, or 5 mM Ca^{2+} without exposure to nsPEFs, shown as mean ± SEM. Traces were vertically offset along the y-axis for improved visual clarity. All measurements originated from the same baseline fluorescence intensity, and the vertical displacement does not reflect differences in absolute fluorescence values. **b.** Bar graph showing the distribution of mean YO-PRO-1 fluorescence intensity in spheroids of RT4 and SV-HUC1 cells as well as PDOs measured 600 seconds after exposure in solutions containing 0 mM, 2 mM, and 5 mM Ca^{2+} (n = 9–11) Error bars represent the minimum to maximum range. Statistical comparisons were performed using Welch's ANOVA with Dunnett's correction for multiple comparisons. For 0 mM Ca^{2+}, W(4, 20.65) = 5.139; for 2 mM Ca^{2+}, W(4, 19.15) = 3.408; and for 5 mM Ca^{2+}, W(4, 15.95) = 5.697. **c.** Bar graph showing the distribution of AUC of YO-PRO-1 fluorescence intensity in spheroids of RT4 and SV-HUC1 cells as well as PDOs measured 600 seconds after exposure (n = 9–11). Statistical comparisons of the AUC of fluorescence between normal urothelial SV-HUC-1 spheroids and cancer RT4 spheroids with PDO were performed using Welch's ANOVA, followed by Dunnett's T3 post hoc test for multiple comparisons: for 0 mM Ca^{2+}, W(4, 28.14) = 28.34, for 2 mM Ca^{2+}, W(4, 20.46) = 38.53 and for 5 mM Ca^{2+}, W(4, 18.09) = 53.60. **d.** Quantification of the area under the curve (AUC) of fluorescence dynamics was performed for spheroids and PDOs under different Ca^{2+} conditions. Bar Graph show the data distribution and mean ± SEM (*n* = 9–11). Statistical comparisons were conducted using Welch's ANOVA, followed by Dunnett's T3 post hoc test to compare 2 mM and 5 mM Ca^{2+} conditions to the 0 mM Ca^{2+}. PDO 154, W(2, 14.96) = 17.49; PDO 319, W(2, 19.74) = 3.87; PDO 270, W(2, 13.19) = 81.94; SV-HUC-1, W(2, 28.10) = 25.21; and RT4, W(2, 16.26) = 69.05. **e**. Linear regression analysis was performed to assess correlations between spheroid size or mean single-cell area within spheroids and total fluorescence intensity. The analysis included data from 45-50 spheroids and PDOs (RT4, SV-HUC-1, PDO 154, PDO 270, and PDO 319) per condition, with each point representing an individual spheroid (n = 45-50). Separate regressions were performed for 0 mM, 2 mM, and 5 mM Ca^{2+} conditions.

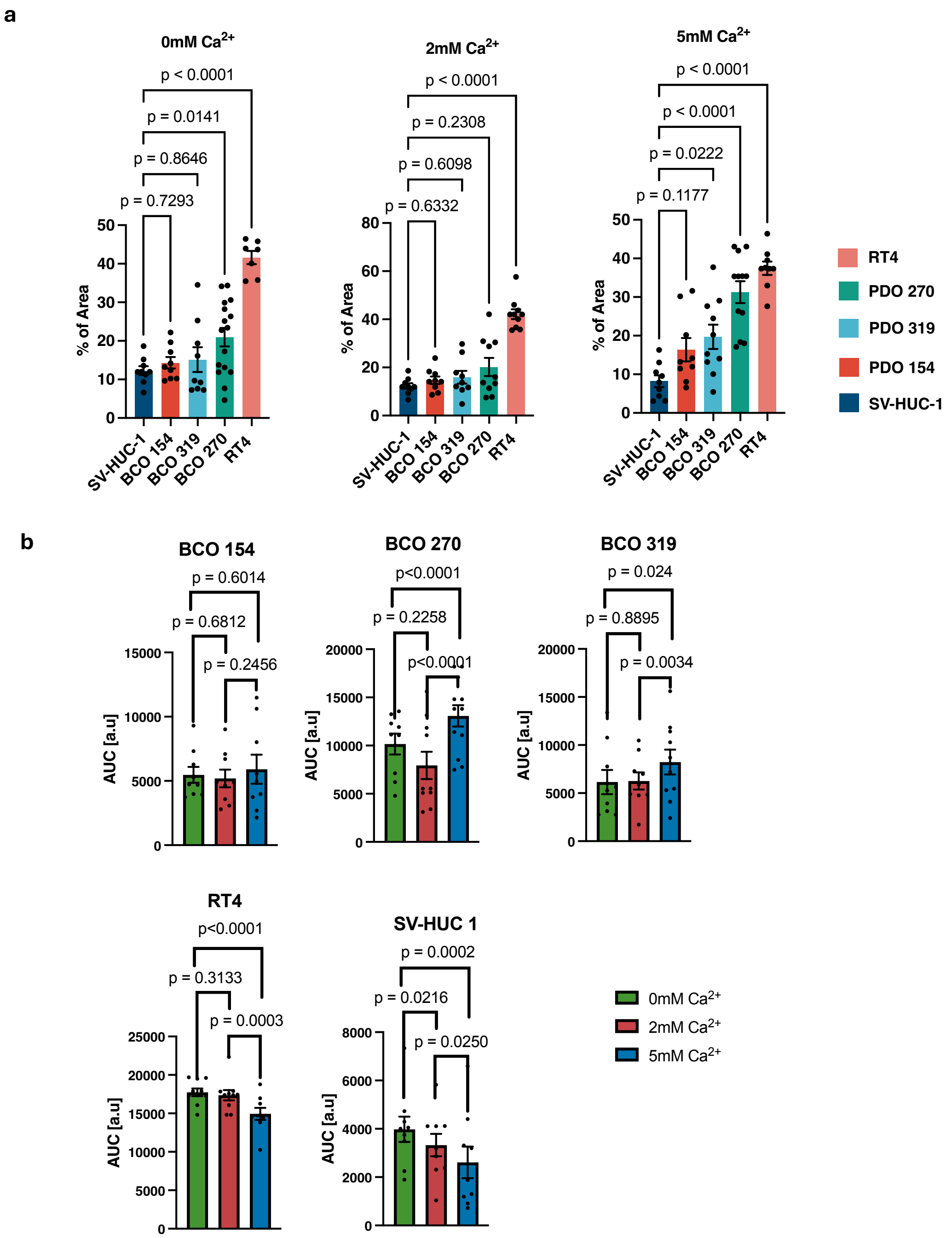

Supplementary Figure S8. Changes in the 2D projected area of spheroids and PDOs following exposure to nsPEFs. a percentage changes in the 2D projected area of spheroids and PDOs (mean ± SEM, n=9-11) 10 minutes after nsPEFs exposure in solutions containing 0 mM, 2 mM, or 5 mM Ca^{2}. Statistical comparison of 2D projected area changes over time were performed using Welch's ANOVA, followed by Dunnett's T3 post hoc test to compare normal urothelial SV-HUC-1 spheroids with cancer RT4 spheroids and PDOs in different Ca^{2+} concentrations: for 0 mM Ca^{2+}, 86,16 (4,000, 19,81) ; for 2 mM Ca^{2+}, 25,92 (4,000, 26,37); and for 5 mM Ca^{2+}, W40,36 (4,000, 21,54). **b.** Comparison of the AUC of 2D projected area dynamics in spheroids and PDOs (mean ± SEM, n = 9–11) was performed across different extracellular Ca^{2+} concentrations. Statistical analysis was conducted using Welch's ANOVA followed by Dunnett's T3 post hoc test. The following Welch's ANOVA test statistics were obtained: SV-HUC-1, W(2, 15.76) = 14.62; BCO 154, W(2, 15.28) = 1.619; BCO 270, W(2, 25.81) = 28.46; BCO 319, W(2, 15.45) = 9.961; and RT4, W(2, 11.51) = 31.19.

a

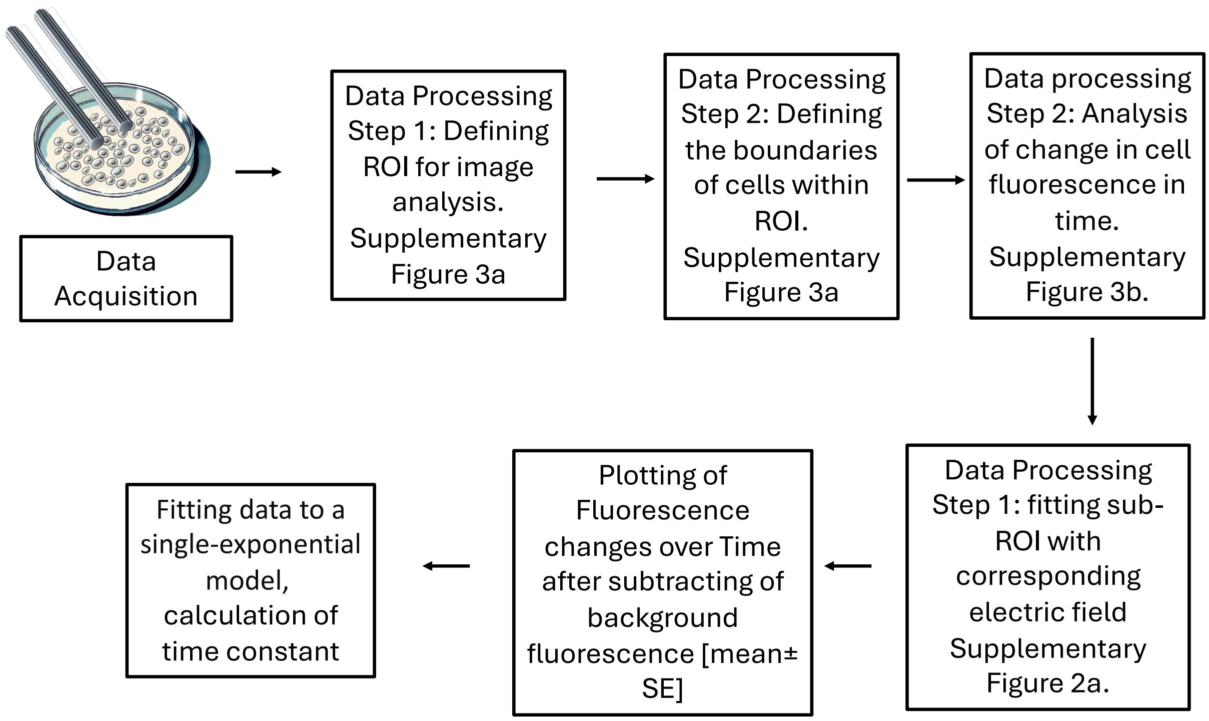

b

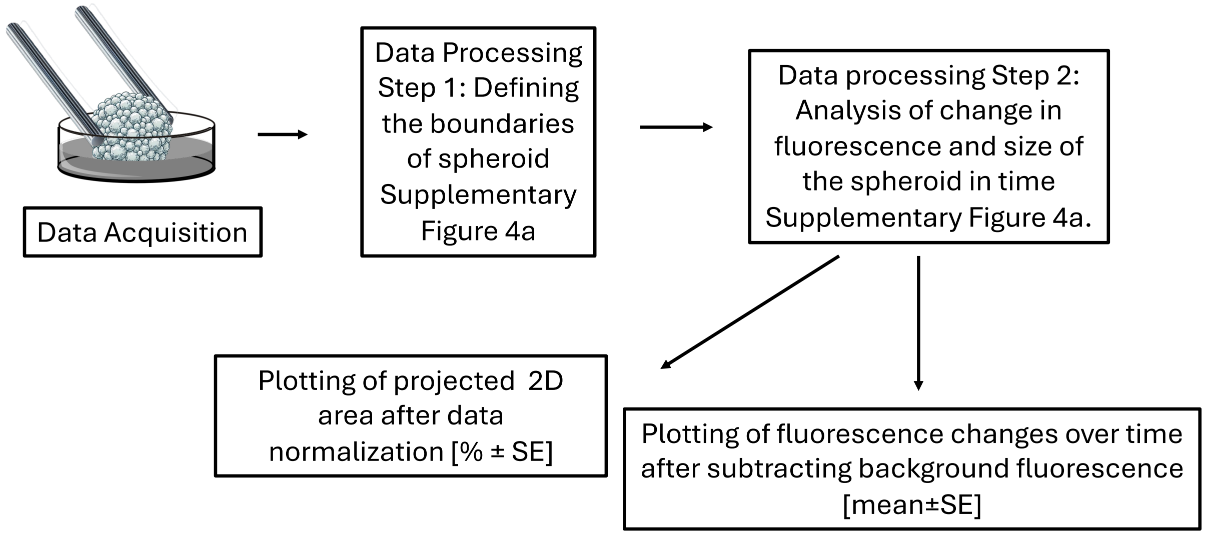

Supplementary Figure S9: Overview of the technical workflow. a. Technical workflow for monolayer electroporation: image acquisition during electroporation in YO-PRO-1 physiological solution, selection of the region of interest (ROI), merging with the simulated electric field intensity map, background correction, fluorescence plotting, and non-linear regression fitting. **b.** Technical workflow for spheroid electroporation: image acquisition during electroporation of a spheroid or organoid in YO-PRO-1 solution, detection of spheroid boundaries, analysis of fluorescence and size changes over time, background correction, and plotting of normalized projected area and fluorescence over time.

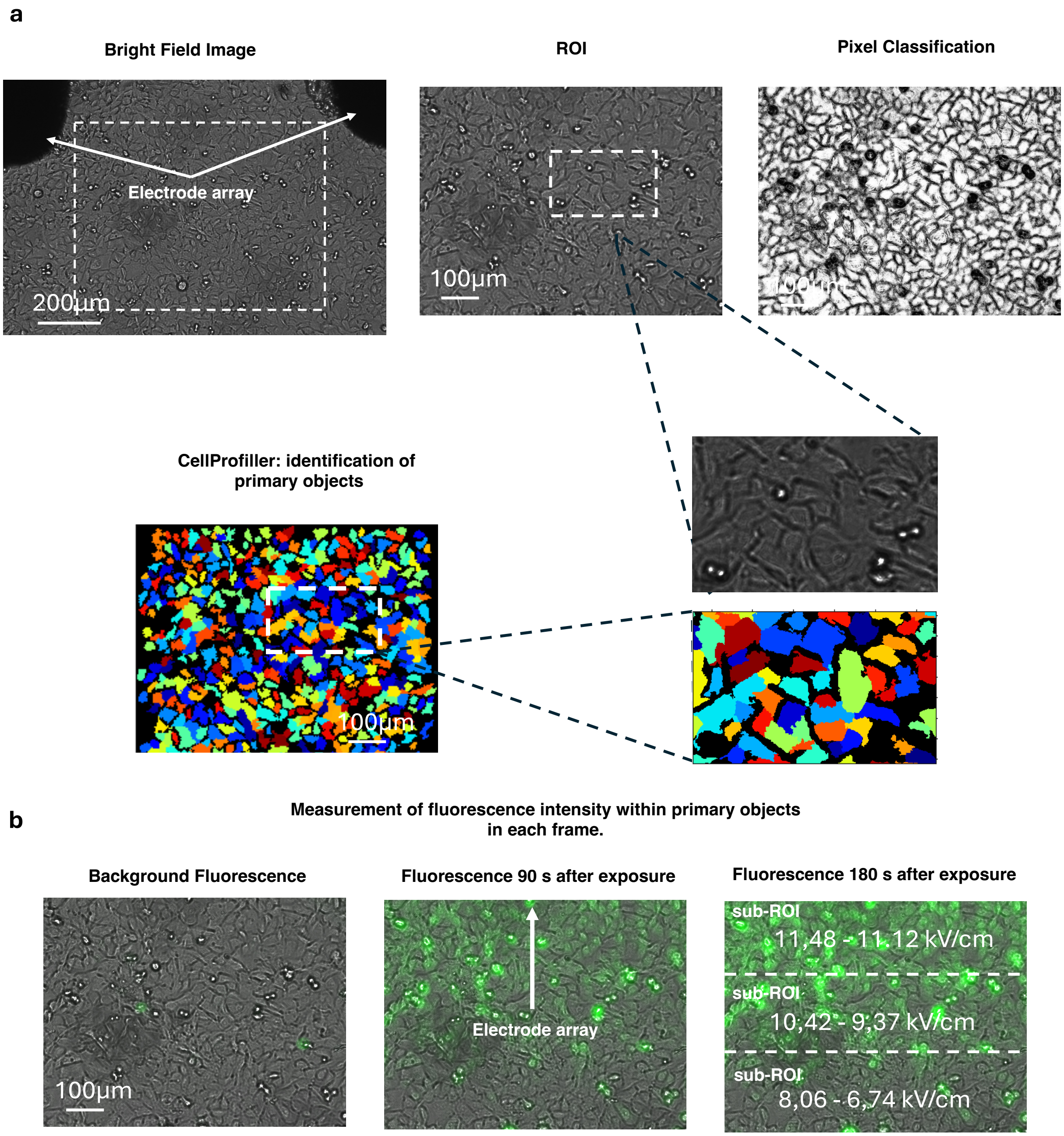

Supplementary Figure S10: Image Analysis Pipeline for Monolayer Electroporation a. After defining the region of interest (ROI) within the monolayer, individual cells were identified using ilastik's machine learning–based pixel classification. The classifier, trained on user-labeled regions, computed multi-scale image features—including edge detection, texture, and intensity—to accurately distinguish cells from the background. **b.** Fluorescence changes were monitored over a 180-second period. Detected primary objects (cells) were classified according to their position within the ROI. Fluorescence signals were then assigned to the corresponding sub-ROIs and averaged across all cells within each sub-region.
