# Supplementary material for: Nanosecond pulsed electric fields induce cell-size-dependent selective permeabilization of urothelial cancer cells
Source: Commun Biol. 2025 Dec 30;9:153. doi: 10.1038/s42003-025-09432-7 (PMC12868620; doi:10.1038/s42003-025-09432-7)
Supplement: Supplementary file 3 — Description of Additional Supplementary Files [file 42003_2025_9432_MOESM3_ESM.pdf]

Description of Additional Supplementary File

File name: Supplementary Data
Description: This file provides the complete numerical source data for all graphical elements in the manuscript. Individual worksheets are labeled according to the corresponding figure or graph panel and contain the datasets used for their preparation.
