# Supplementary material for: Nanosecond pulsed electric fields induce cell-size-dependent selective permeabilization of urothelial cancer cells
Source: Commun Biol. 2025 Dec 30;9:153. doi: 10.1038/s42003-025-09432-7 (PMC12868620; doi:10.1038/s42003-025-09432-7)
Supplement: Supplementary file 5 — Reporting Summary [file 42003_2025_9432_MOESM5_ESM.pdf]

nature portfolio

Corresponding author(s): Aleksander Kielbik

Last updated by author(s): 30.10.2025

Reporting Summary

Nature Portfolio wishes to improve the reproducibility of the work that we publish. This form provides structure for consistency and transparency in reporting. For further information on Nature Portfolio policies, see our Editorial Policies and the Editorial Policy Checklist.

Statistics

For all statistical analyses, confirm that the following items are present in the figure legend, table legend, main text, or Methods section.

n/a	Confirmed	
☐	☒	The exact sample size (*n*) for each experimental group/condition, given as a discrete number and unit of measurement
☐	☒	A statement on whether measurements were taken from distinct samples or whether the same sample was measured repeatedly
☐	☒	The statistical test(s) used AND whether they are one- or two-sided<br>*Only common tests should be described solely by name; describe more complex techniques in the Methods section.*
☒	☐	A description of all covariates tested
☐	☒	A description of any assumptions or corrections, such as tests of normality and adjustment for multiple comparisons
☐	☒	A full description of the statistical parameters including central tendency (e.g. means) or other basic estimates (e.g. regression coefficient) AND variation (e.g. standard deviation) or associated estimates of uncertainty (e.g. confidence intervals)
☐	☒	For null hypothesis testing, the test statistic (e.g. *F*, *t*, *r*) with confidence intervals, effect sizes, degrees of freedom and *P* value noted<br>*Give P values as exact values whenever suitable.*
☒	☐	For Bayesian analysis, information on the choice of priors and Markov chain Monte Carlo settings
☒	☐	For hierarchical and complex designs, identification of the appropriate level for tests and full reporting of outcomes
☒	☐	Estimates of effect sizes (e.g. Cohen's *d*, Pearson's *r*), indicating how they were calculated

Our web collection on statistics for biologists contains articles on many of the points above.

Software and code

Policy information about availability of computer code

Data collection	no software was used
Data analysis	no software was used

For manuscripts utilizing custom algorithms or software that are central to the research but not yet described in published literature, software must be made available to editors and reviewers. We strongly encourage code deposition in a community repository (e.g. GitHub). See the Nature Portfolio guidelines for submitting code & software for further information.

Data

Policy information about availability of data

All manuscripts must include a data availability statement. This statement should provide the following information, where applicable:

- Accession codes, unique identifiers, or web links for publicly available datasets
- A description of any restrictions on data availability
- For clinical datasets or third party data, please ensure that the statement adheres to our policy

datasets are available from the corresponding author upon reasonable request.

Research involving human participants, their data, or biological material

Policy information about studies with human participants or human data. See also policy information about sex, gender (identity/presentation), and sexual orientation and race, ethnicity and racism.

Reporting on sex and gender	The sex of the cell donors for urothelial organoids has been included in the report. This information was obtained from medical records following patient consent.
Reporting on race, ethnicity, or other socially relevant groupings	no socialy relevant catogorisation was used in the manuscript
Population characteristics	Clinical data of the cell donors for urothelial organoids are included in the supplementary data. This information was obtained from medical records with informed patient consent.
Recruitment	Patients diagnosed with bladder cancer at the University Clinic in Tübingen were asked to serve as cell donors following informed consent. Donors may represent a specific demographic, clinical, or molecular subgroup of bladder cancer, influenced by regional, genetic, environmental, or institutional treatment factors.
Ethics oversight	This study was approved by the Scientific Board at the University of Tuebingen

Note that full information on the approval of the study protocol must also be provided in the manuscript.

Field-specific reporting

Please select the one below that is the best fit for your research. If you are not sure, read the appropriate sections before making your selection.

☒ Life sciences ☐ Behavioural & social sciences ☐ Ecological, evolutionary & environmental sciences

For a reference copy of the document with all sections, see nature.com/documents/nr-reporting-summary-flat.pdf

Life sciences study design

All studies must disclose on these points even when the disclosure is negative.

Sample size	No sample size calculation was performed. The sample size was determined based on practical considerations, such as resource availability and previous similar studies.
Data exclusions	No data were excluded from analysis
Replication	Data from all experiments include both biological and technical replicates
Randomization	Samples—including cell lines, primary cells, and electroporation conditions—were randomized
Blinding	No blinding was performed

Behavioural & social sciences study design

All studies must disclose on these points even when the disclosure is negative.

Study description	The acquired data are quantitative and asses biological efficacy and mechanism of PEFs on urothelial cells
Research sample	Bladder cell lines and primary cancer cells from patients diagnosed with bladder cancer at the University Clinic in Tübingen
Sampling strategy	No sample size calculation was performed. The sample size was determined based on practical considerations, such as resource availability and previous similar studies.
Data collection	Data were collected by the research team and recorded electronically
Timing	Data were collected over the course of approximately one year during the study period
Data exclusions	No data were excluded from analysis
Non-participation	no participants dropped out or declined participation
Randomization	Samples—including cell lines, primary cells, and electroporation conditions—were randomized

Ecological, evolutionary & environmental sciences study design

All studies must disclose on these points even when the disclosure is negative.

Study description	The acquired data are quantitative, data from all experiments include both biological and technical replicates
Research sample	Bladder cell lines and primary cancer cells from patients diagnosed with bladder cancer at the University Clinic in Tübingen
Sampling strategy	No sample size calculation was performed. The sample size was determined based on practical considerations, such as resource availability and previous similar studies.
Data collection	Data were collected by the research team and recorded electronically
Timing and spatial scale	Data were collected over the course of approximately one year during the study period
Data exclusions	No data were excluded from analysis
Reproducibility	The experimental design incorporated both biological and technical replicates. Findings were confirmed across four urothelial cancer cell lines and primary organoids derived from patient samples.
Randomization	Samples—including cell lines, primary cells, and electroporation conditions—were randomized
Blinding	No blinding was performed

Did the study involve field work? ☐ Yes ☒ No

Field work, collection and transport

Field conditions	No field work was performed
Location	No field work was performed
Access & import/export	No field work was performed
Disturbance	No field work was performed

Reporting for specific materials, systems and methods

We require information from authors about some types of materials, experimental systems and methods used in many studies. Here, indicate whether each material, system or method listed is relevant to your study. If you are not sure if a list item applies to your research, read the appropriate section before selecting a response.

Materials & experimental systems

n/a	Involved in the study	
☒	☐	Antibodies
☐	☒	Eukaryotic cell lines
☒	☐	Palaeontology and archaeology
☒	☐	Animals and other organisms
☐	☒	Clinical data
☒	☐	Dual use research of concern
☒	☐	Plants

Methods

n/a	Involved in the study	
☒	☐	ChIP-seq
☒	☐	Flow cytometry
☒	☐	MRI-based neuroimaging

Antibodies

Antibodies used	No Antibodies were used in the Study
Validation	No Antibodies were used in the Study

Eukaryotic cell lines

Policy information about cell lines and Sex and Gender in Research

Cell line source(s)	ATCC, CELLnTEC
Authentication	Cells were acquired from ATACC and CELLnTEC and utilized directly following acquisition
Mycoplasma contamination	All cell lines were tested negative for Mycoplasma contamination
Commonly misidentified lines (See ICLAC register)	no commonly misidentified lines have been used in the study

Palaeontology and Archaeology

Specimen provenance	Primary urothelial tumor cells were obtained from five patients undergoing transurethral resection or cystectomy for bladder cancer at the University Hospital Tübingen. The use of human tissue was approved by the institutional ethics committee (approval no. 804/2020/B02), and written informed consent was obtained from all participants.
Specimen deposition	The primary urothelial cancer cells were deposited in the Department of Urology laboratory at the University Hospital Tübingen
Dating methods	Data were collected by the research team and recorded electronically

☐ Tick this box to confirm that the raw and calibrated dates are available in the paper or in Supplementary Information.

Ethics oversight	The use of human tissue was approved by the institutional ethics committee (approval no. 804/2020/B02), and written informed consent was obtained from all participants.

Note that full information on the approval of the study protocol must also be provided in the manuscript.

Animals and other research organisms

Policy information about studies involving animals; ARRIVE guidelines recommended for reporting animal research, and Sex and Gender in Research

Laboratory animals	this study did not involved laboratory animals
Wild animals	this study did not involved wild animals
Reporting on sex	The sex of the cell donors for urothelial organoids has been included in the report. This information was obtained from medical records following patient consent.
Field-collected samples	this study did not involved field-collected samples
Ethics oversight	This study was approved by the Scientific Board at the University of Tuebingen

Note that full information on the approval of the study protocol must also be provided in the manuscript.

Clinical data

Policy information about clinical studies

All manuscripts should comply with the ICMJE guidelines for publication of clinical research and a completed CONSORT checklist must be included with all submissions.

Clinical trial registration	No clinical trial has been performed
Study protocol	The study protocol is described in the Methods section
Data collection	Clinical data of donors are provided in the supplementary data.
Outcomes	The outcome is reported in the manuscript

Dual use research of concern

Policy information about dual use research of concern

Hazards

Could the accidental, deliberate or reckless misuse of agents or technologies generated in the work, or the application of information presented in the manuscript, pose a threat to:

No	Yes	
☒	☐	Public health
☒	☐	National security
☒	☐	Crops and/or livestock
☒	☐	Ecosystems
☒	☐	Any other significant area

Experiments of concern

Does the work involve any of these experiments of concern:

No	Yes	
☒	☐	Demonstrate how to render a vaccine ineffective
☒	☐	Confer resistance to therapeutically useful antibiotics or antiviral agents
☒	☐	Enhance the virulence of a pathogen or render a nonpathogen virulent
☒	☐	Increase transmissibility of a pathogen
☒	☐	Alter the host range of a pathogen
☒	☐	Enable evasion of diagnostic/detection modalities
☒	☐	Enable the weaponization of a biological agent or toxin
☒	☐	Any other potentially harmful combination of experiments and agents

Plants

Seed stocks	this study did not involved seeds or plant material
Novel plant genotypes	this study did not involved plant genotypes
Authentication	this study did not involved seeds or plant material

ChIP-seq

Data deposition

☐ Confirm that both raw and final processed data have been deposited in a public database such as GEO.

☐ Confirm that you have deposited or provided access to graph files (e.g. BED files) for the called peaks.

Data access links<br>*May remain private before publication.*	the study did not involved ChIP-seq
Files in database submission	the study did not involved ChIP-seq
Genome browser session<br>(e.g. UCSC)	the study did not involved ChIP-seq

Methodology

Replicates	the study did not involved ChIP-seq
Sequencing depth	the study did not involved ChIP-seq
Antibodies	the study did not involved ChIP-seq
Peak calling parameters	the study did not involved ChIP-seq

Data quality	the study did not involved ChIP-seq
Software	the study did not involved ChIP-seq

Flow Cytometry

Plots

Confirm that:

☐ The axis labels state the marker and fluorochrome used (e.g. CD4-FITC).

☐ The axis scales are clearly visible. Include numbers along axes only for bottom left plot of group (a 'group' is an analysis of identical markers).

☐ All plots are contour plots with outliers or pseudocolor plots.

☐ A numerical value for number of cells or percentage (with statistics) is provided.

Methodology

Sample preparation	this study did not involved flow cytometry
Instrument	this study did not involved flow cytometry
Software	this study did not involved flow cytometry
Cell population abundance	this study did not involved flow cytometry
Gating strategy	this study did not involved flow cytometry

☐ Tick this box to confirm that a figure exemplifying the gating strategy is provided in the Supplementary Information.

Magnetic resonance imaging

Experimental design

Design type	this study did not involved Magnetic resonance imaging
Design specifications	this study did not involved Magnetic resonance imaging
Behavioral performance measures	this study did not involved Magnetic resonance imaging

Acquisition

Imaging type(s)	this study did not involved Magnetic resonance imaging
Field strength	this study did not involved Magnetic resonance imaging
Sequence & imaging parameters	this study did not involved Magnetic resonance imaging
Area of acquisition	this study did not involved Magnetic resonance imaging

Diffusion MRI ☐ Used ☒ Not used

Preprocessing

Preprocessing software	this study did not involved Magnetic resonance imaging
Normalization	this study did not involved Magnetic resonance imaging
Normalization template	this study did not involved Magnetic resonance imaging
Noise and artifact removal	this study did not involved Magnetic resonance imaging
Volume censoring	this study did not involved Magnetic resonance imaging

Statistical modeling & inference

Model type and settings	this study did not involved Magnetic resonance imaging

Effect(s) tested | this study did not involved Magnetic resonance imaging

Specify type of analysis: ☐ Whole brain ☐ ROI-based ☐ Both

Statistic type for inference (See Eklund et al. 2016) | this study did not involved Magnetic resonance imaging

Correction | this study did not involved Magnetic resonance imaging

Models & analysis

n/a	Involved in the study
☒	☐ Functional and/or effective connectivity
☒	☐ Graph analysis
☒	☐ Multivariate modeling or predictive analysis

Functional and/or effective connectivity | this study did not involved Magnetic resonance imaging

Graph analysis | this study did not involved Magnetic resonance imaging

Multivariate modeling and predictive analysis | this study did not involved Magnetic resonance imaging
